# Activation of the VPAC2 Receptor Impairs Axon Outgrowth and Decreases Dendritic Arborization in Mouse Cortical Neurons by a PKA-Dependent Mechanism

**DOI:** 10.3389/fnins.2020.00521

**Published:** 2020-06-04

**Authors:** Shuto Takeuchi, Takuya Kawanai, Ryosuke Yamauchi, Lu Chen, Tatsunori Miyaoka, Mei Yamada, Satoshi Asano, Atsuko Hayata-Takano, Takanobu Nakazawa, Koji Yano, Naotaka Horiguchi, Shinsaku Nakagawa, Kazuhiro Takuma, James A. Waschek, Hitoshi Hashimoto, Yukio Ago

**Affiliations:** ^1^Laboratory of Molecular Neuropharmacology, Graduate School of Pharmaceutical Sciences, Osaka University, Suita, Japan; ^2^Laboratory of Biopharmaceutics, Graduate School of Pharmaceutical Sciences, Osaka University, Suita, Japan; ^3^Department of Cellular and Molecular Pharmacology, Graduate School of Biomedical and Health Sciences, Hiroshima University, Hiroshima, Japan; ^4^Molecular Research Center for Children’s Mental Development, United Graduate School of Child Development, Osaka University, Kanazawa University, Hamamatsu University School of Medicine, Chiba University and University of Fukui, Suita, Japan; ^5^Department of Pharmacology, Graduate School of Dentistry, Osaka University, Suita, Japan; ^6^Neuroscience Department, Drug Discovery and Disease Research Laboratory, Shionogi Pharmaceutical Research Center, Shionogi & Co., Ltd., Toyonaka, Japan; ^7^Laboratory of Innovative Food Science, Graduate School of Pharmaceutical Sciences, Osaka University, Suita, Japan; ^8^Global Center for Medical Engineering and Informatics, Osaka University, Suita, Japan; ^9^Department of Psychiatry and Biobehavioral Sciences, David Geffen School of Medicine, Semel Institute for Neuroscience and Human Behavior, University of California, Los Angeles, Los Angeles, CA, United States; ^10^Division of Bioscience, Institute for Datability Science, Osaka University, Suita, Japan; ^11^Transdimensional Life Imaging Division, Institute for Open and Transdisciplinary Research Initiatives, Osaka University, Suita, Japan; ^12^Department of Molecular Pharmaceutical Science, Graduate School of Medicine, Osaka University, Suita, Japan

**Keywords:** VPAC2 receptor, psychiatric disorders, cortical neurons, axon, dendrite

## Abstract

Clinical studies have shown that microduplications at 7q36.3, containing *VIPR2*, confer significant risk for schizophrenia and autism spectrum disorder (ASD). *VIPR2* gene encodes the VPAC2 receptor for vasoactive intestinal peptide (VIP) and pituitary adenylate cyclase-activating polypeptide (PACAP). Lymphocytes from patients with these mutations exhibited higher *VIPR2* gene expression and VIP-induced cAMP responsiveness, but mechanisms by which overactive VPAC2 signaling may lead to these psychiatric disorders are unknown. We have previously found that repeated administration of a selective VPAC2 receptor agonist Ro25-1553 in the mouse during early postnatal development caused synaptic alterations in the prefrontal cortex and sensorimotor gating deficits. In this study, we aimed to clarify the effects of VPAC2 receptor activation on neurite outgrowth in cultured primary mouse cortical neurons. Ro25-1553 and VIP caused reductions in total numbers and lengths of both neuronal dendrites and axons, while PACAP38 facilitated elongation of dendrites, but not axons. These effects of Ro25-1553 and VIP were blocked by a VPAC2 receptor antagonist PG99-465 and abolished in VPAC2 receptor-deficient mice. Additionally, Ro25-1553-induced decreases in axon and dendritic outgrowth in wild-type mice were blocked by a protein kinase A (PKA) inhibitor H89, but not by a PKC inhibitor GF109203X or a mitogen-activated protein kinase (MAPK) kinase (MEK) inhibitor U0126. PACAP38- induced facilitation of dendritic outgrowth was blocked by U0126. These results suggest that activation of the VPAC2 receptor impairs neurite outgrowth and decreases branching of cortical neurons by a PKA-dependent mechanism. These findings also imply that the *VIPR2*-linkage to mental health disorders may be due in part to deficits in neuronal maturation induced by VPAC2 receptor overactivation.

## Introduction

Accumulating evidence indicates that a number of rare copy number variants (CNVs), including both deletions and duplications, have been strongly associated with schizophrenia and neurodevelopmental disorders such as autism spectrum disorder (ASD) (Sullivan et al., 2012; Foley et al., 2017; Deshpande and Weiss, 2018; Vicari et al., 2019). Among the most highly penetrant genetic risk factors for neuropsychiatric disorders, clinical studies have shown that microduplications at 7q36.3, containing *VIPR2*, confer significant risk for schizophrenia (Levinson et al., 2011; Vacic et al., 2011; Yuan et al., 2014; Li et al., 2016; Marshall et al., 2017) and ASD (Vacic et al., 2011; Firouzabadi et al., 2017). *VIPR2* encodes VPAC2, a seven transmembrane heterotrimeric G protein-coupled receptor (Gs) that binds two homologous neuropeptides with high affinity, vasoactive intestinal peptide (VIP) and pituitary adenylate cyclase-activating polypeptide (PACAP). Lymphocytes from patients with these microduplications exhibited higher *VIPR2* gene expression and VIP responsiveness (cAMP induction) (Vacic et al., 2011), demonstrating the functional significance of the microduplications. Additionally, the blood concentration of VIP, but not PACAP, was higher in children with ASD compared to healthy control subjects (Nelson et al., 2001). These suggest that overactivation of the VPAC2 receptor signaling is involved in the etiology of schizophrenia and ASD. We previously found that repeated administration of the selective VPAC2 receptor agonist Ro25-1553 in the mouse during early postnatal development caused prepulse inhibition deficits and reductions in synaptic proteins synaptophysin and postsynaptic density protein 95 (PSD-95) in the prefrontal cortex, but not in the hippocampus (Ago et al., 2015). Recently, Tian et al. (2019) have developed a conditional human *VIPR2* CNV bacterial artificial chromosome (BAC) transgenic (h*VIPR2*-BAC tg) mouse model of *VIPR2* CNV, and they reported that h*VIPR2*-BAC tg mice showed cognitive, sensorimotor gating, and social behavioral deficits and decrease in the complexity of dendritic arborization of the striatal spiny projection neurons. Additionally, VPAC2 receptor knockout mice have abnormal dendritic morphology of the prefrontal cortex neurons, but not basolateral amygdala neurons (Ago et al., 2017). These findings suggest that the VPAC2 receptor plays an important role in the regulation of the dendritic morphology and overactivation of the VPAC2 receptor might impair neural development in the brain.

Both PACAP and VIP have been known to regulate cell proliferation, differentiation, survival, maturation, neurite outgrowth, and expression of trophic factors (see review: Waschek, 1996, 2002; Muller et al., 2006; Falluel-Morel et al., 2007; Hill, 2007; Vaudry et al., 2009; Harmar et al., 2012). In particular, our and other studies have shown that PACAP and VIP promote neurite outgrowth in sympathetic precursors (Lu et al., 1998; DiCicco-Bloom et al., 2000; Nicot and DiCicco-Bloom, 2001; Suh et al., 2001), pheochromocytoma PC12 cells (Deutsch and Sun, 1992; Hernandez et al., 1995; Lazarovici et al., 1998; Vaudry et al., 2002; Sakai et al., 2004; Manecka et al., 2013), neuroblastoma NB-OK, Neuro2a and SH-SY5Y cells (Deutsch et al., 1993; Héraud et al., 2004; Monaghan et al., 2008; Kambe and Miyata, 2012) and primary cultured hippocampal neurons (Leemhuis et al., 2007; Kambe and Miyata, 2012; Ogata et al., 2015), cerebellar granule cells (Gonzalez et al., 1997; Falluel-Morel et al., 2005), dorsal root ganglion cells (White and Mansfield, 1996; White et al., 2000), and trigeminal ganglion cells (Fukiage et al., 2007). In contrast, both PACAP and VIP inhibited the induction of dendritic growth by bone morphogenetic protein-7 (BMP-7) in primary cultured sympathetic neurons (Drahushuk et al., 2002). This highlights a hierarchal reversal of action that may occur in the presence of a patterning molecule. Opposing activities of PACAP on neural precursors have also been observed in other contexts. For instance, the action of PACAP on embryonic rat cortical precursors switches from antimitotic to promitotic depending on the presence or absence of specific PACAP receptor splice variants (Nicot and DiCicco-Bloom, 2001). The switch to promitotic action appeared to occur by a mechanism that involves recruitment of the phospholipase C pathway. Overall, these findings suggest that neuropeptides PACAP and VIP can exhibit tissue- or cell-type-specific effects due to the expression level and pattern of the PACAP receptor subtypes and splice variants, and the presence or absence of growth and patterning factors. In most cases, however, the precise receptors and intracellular signaling mechanisms that account for this remain unknown.

Here we aimed to investigate the effects of the VPAC2 receptor activation by using Ro25-1553 and VPAC2 receptor-null mice on axon and dendritic outgrowth in primary cultured mouse cortical neurons. We also compare the effects of Ro25-1553 with those of VIP and PACAP. Furthermore, we examined the intracellular signaling pathways involved in the effects of Ro25-1553 and PACAP.

## Animals and Methods

### Animals and Materials

**FIGURE 1:**
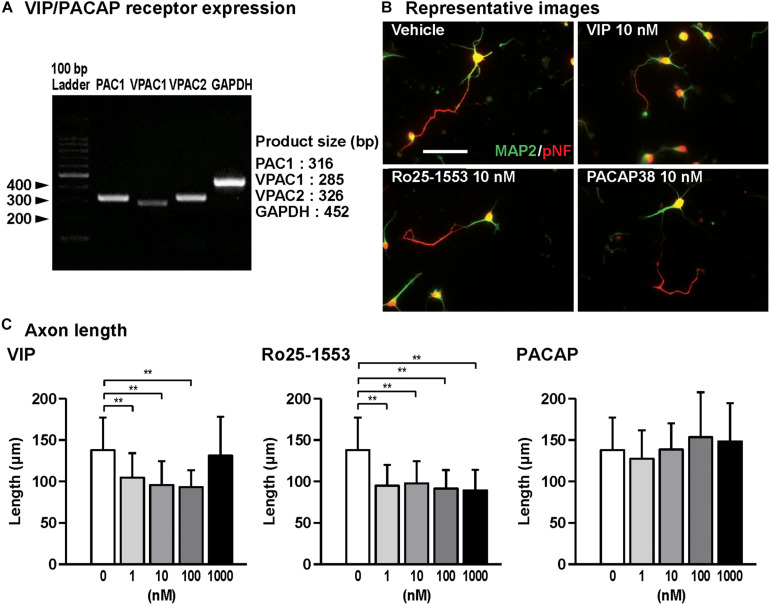
Effects of Ro25-1553, VIP, and PACAP on axon outgrowth in cultured cortical neurons. **(A)** RT-PCR analysis showed that all VIP/PACAP receptors PAC1, VPAC1, and VPAC2 were expressed in primary cultured cortical neurons. **(B)** Representative pNF- and MAP2-immunostained images of cultured cortical neurons are shown. Cells were cultured with Ro25-1553 (10 nM), VIP (10 nM), or PACAP38 (10 nM) for 3 days *in vitro* and double-immunostained for pNF (red) and MAP2 (green). Scale bar, 50 μm. **(C)** Quantitative analysis of changes in axon length was shown. VIP (*F*_4_,_215_ = 16.542, *P* < 0.0001) and Ro25-1553 (*F*_4,215_ = 25.257, *P* < 0.0001), but not PACAP (*F*_4,215_ = 2.383, *P* > 0.05), reduced the axon length. Values represent mean ± SD of 40–60 neurons from three independent experiments. **P < 0.01 vs. control.

**FIGURE 2:**
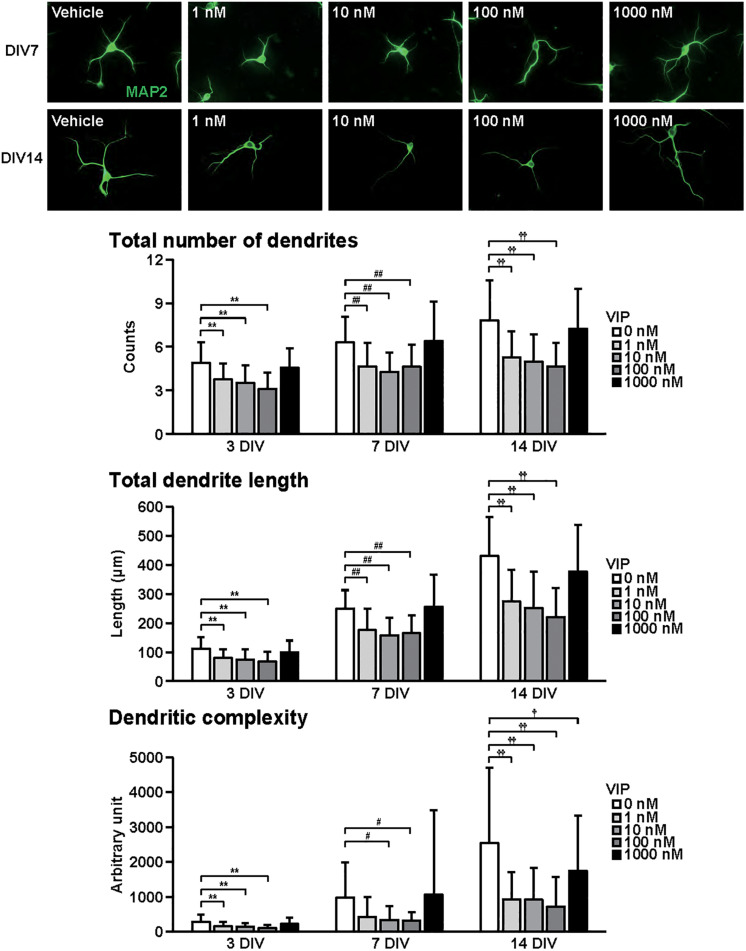
Effects of VIP on dendritic outgrowth in cultured cortical neurons. Representative MAP2 (green)-immunostained images of neurons cultured with VIP (1–1000 nM) for 7 to 14 days *in vitro* (DIV) are shown. Quantitative analysis of dendritic morphology at 3 (Figure 1B), 7, and 14 DIV revealed that VIP significantly decreased the total numbers (main effects: *F*_2,805_ = 75.487, *P* < 0.0001 for time, *F*_4,805_ = 52.424, *P* < 0.0001 for treatment; interaction: *F*_8,805_ = 2.556, *P* < 0.01) and length (main effects: *F*_2,805_ = 374.760, *P* < 0.0001 for time, *F*_4,805_ = 51.272, *P* < 0.0001 for treatment; interaction: *F*_8,805_ = 8.563, *P* < 0.0001) of dendrites, and dendritic complexity (main effects: *F*_2,805_ = 76.423, *P* < 0.0001 for time, *F*_4,805_ = 20.616, *P* < 0.0001 for treatment; interaction: *F*_8,805_ = 5.856, *P* < 0.0001). Values represent mean ± SD of 40–60 neurons from three independent experiments. **P < 0.01, ^#^*P* < 0.05, ^##^*P* < 0.01, ^†^*P* < 0.05, ^††^*P* < 0.01 vs. control.

**FIGURE 3:**
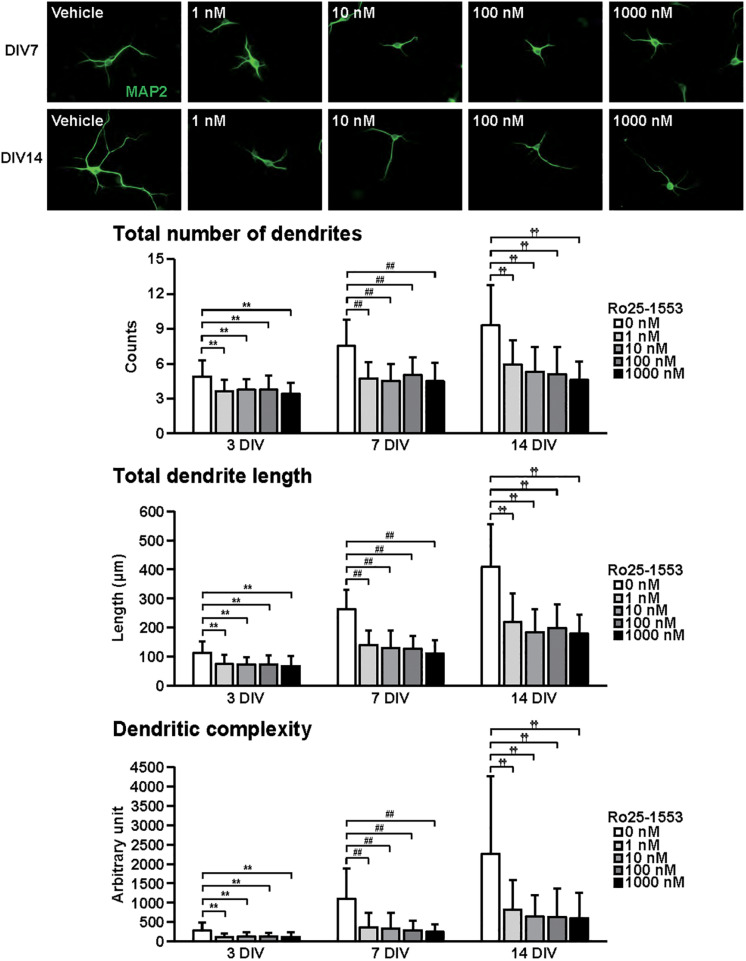
Effects of Ro25-1553 on dendritic outgrowth in cultured cortical neurons. Representative MAP2 (green)-immunostained images of neurons cultured with Ro25-1553 (1–1000 nM) for 7 to 14 days *in vitro* (DIV) are shown. Quantitative analysis of dendritic morphology at 3 (Figure 1B), 7, and 14 DIV revealed that Ro25-1553 significantly decreased the total numbers (main effects: *F*_2,805_ = 83.382, *P* < 0.0001 for time, *F*_4,805_ = 75.612, *P* < 0.0001 for treatment; interaction: *F*_8,805_ = 7.531, *P* < 0.0001) and length (main effects: *F*_2,805_ = 323.212, *P* < 0.0001 for time, *F*_4,805_ = 122.621, *P* < 0.0001 for treatment; interaction: *F*_8,805_ = 17.981, *P* < 0.0001) of dendrites, and dendritic complexity (main effects: *F*_2,805_ = 91.541, *P* < 0.0001 for time, *F*_4,805_ = 49.193, *P* < 0.0001 for treatment; interaction: *F*_8,805_ = 11.777, *P* < 0.0001). Values represent mean ± SD of 40–60 neurons from three independent experiments. **P < 0.01, ^##^*P* < 0.01, ^††^*P* < 0.01 vs. control.

**FIGURE 4:**
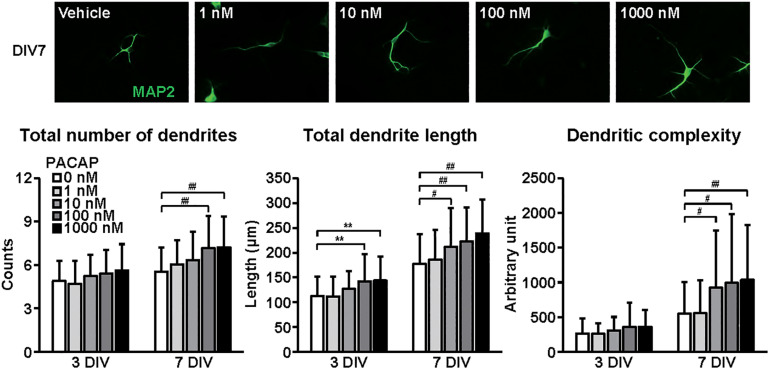
Effects of PACAP on dendritic outgrowth in cultured cortical neurons. Representative MAP2 (green)-immunostained images of neurons cultured with PACAP (1–1000 nM) for 7 days *in vitro* (DIV) are shown. Quantitative analysis of dendritic morphology at 3 (Figure 1B) and 7 DIV revealed that PACAP dose-dependently increased the total numbers (main effects: *F*_1,510_ = 63.825, *P* < 0.0001 for time, *F*_4,510_ = 9.171, *P* < 0.0001 for treatment; interaction: *F*_4,510_ = 1.661, *P* > 0.05) and length (main effects: *F*_1,510_ = 231.606, *P* < 0.0001 for time, *F*_4,510_ = 12.279, *P* < 0.0001 for treatment; interaction: *F*_4,510_ = 0.957, *P* > 0.05) of dendrites, and dendritic complexity (main effects: *F*_1,510_ = 93.016, *P* < 0.0001 for time, *F*_4,510_ = 6.279, *P* < 0.0001 for treatment; interaction: *F*_4,510_ = 2.822, *P* < 0.05). Values represent mean ± SD of 40–60 neurons from three independent experiments. **P < 0.01, ^#^*P* < 0.05, ^##^*P* < 0.01 vs. control.

**FIGURE 5:**
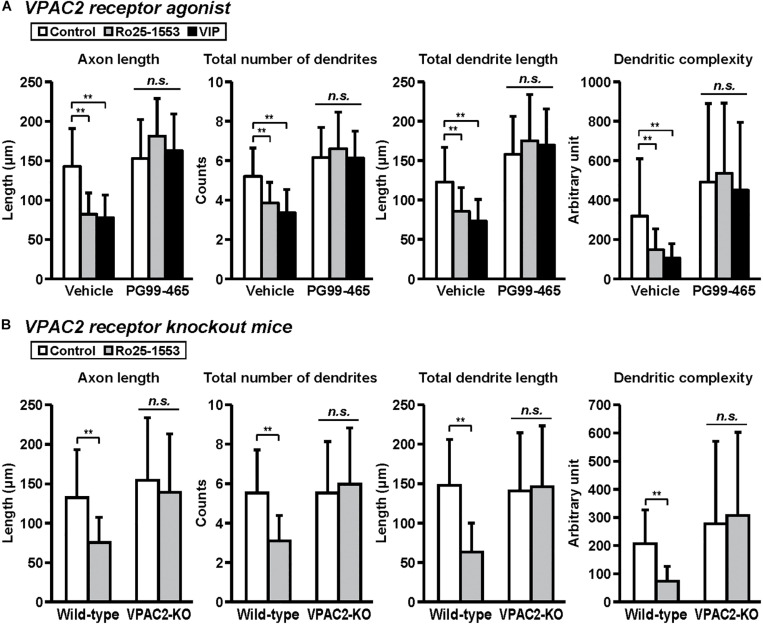
VPAC2 receptor-mediated reductions in axon and dendritic outgrowth in cultured cortical neurons. Primary cortical neurons were cultured with Ro25-1553 (10 nM) or VIP (10 nM) for 3 days *in vitro* and double-immunostained for pNF and MAP2. **(A)** PG99-465 (100 nM) was treated 30 min before the treatment with Ro25-1553 or VIP. PG99-465 blocked Ro25-1553- and VIP-induced reductions in axon length (main effects: *F*_2,215_ = 8.338, *P* < 0.001 for Ro25-1553/VIP, *F*_1,215_ = 129.957, *P* < 0.0001 for PG99-465; interaction: *F*_2,215_ = 24.711, *P* < 0.0001), total numbers (main effects: *F*_2,215_ = 8.231, *P* < 0.001 for Ro25-1553/VIP, *F*_1,215_ = 127.826, *P* < 0.0001 for PG99-465; interaction: *F*_2,215_ = 10.525, *P* < 0.0001) and length (main effects: *F*_2,215_ = 3.701, *P* < 0.05 for Ro25-1553/VIP, *F*_1,215_ = 160.683, *P* < 0.0001 for PG99-465; interaction: *F*_2,215_ = 11.789, *P* < 0.0001) of dendrites, and dendritic complexity (main effects: *F*_2,215_ = 3.744, *P* < 0.05 for Ro25-1553/VIP, *F*_1,215_ = 61.073, *P* < 0.0001 for PG99-465; interaction: *F*_2,215_ = 3.003, *P* < 0.05). **(B)** Primary cortical neurons were prepared from VPAC2 receptor knockout (VPAC2-KO) mice and littermate wild-type mice. Ro25-1553-induced reductions in axon length (main effects: *F*_1,156_ = 12.655 *P* < 0.001 for treatment, *F*_1,156_ = 17.717, *P* < 0.0001 for genotype; interaction: *F*_1,156_ = 4.182, *P* < 0.05), total numbers (main effects: *F*_1,156_ = 7.274, *P* < 0.01 for treatment, *F*_1,156_ = 15.414, *P* < 0.001 for genotype; interaction: *F*_1,156_ = 15.414, *P* < 0.001) and length (main effects: *F*_1,156_ = 15.814, *P* < 0.001 for treatment, *F*_1,156_ = 14.262, *P* < 0.001 for genotype; interaction: *F*_1,156_ = 20.342, *P* < 0.0001) of dendrites, and dendritic complexity (main effects: *F*_1,156_ = 2.175, *P* > 0.05 for treatment, *F*_1,156_ = 19.468, *P* < 0.0001 for genotype; interaction: *F*_1,156_ = 5.576, *P* < 0.05) were abolished in cortical neurons derived from VPAC2-KO mice. Values represent mean ± SD of 40 neurons from three independent experiments. **P < 0.01 vs. control, *n.s.*; not significant.

**FIGURE 6:**
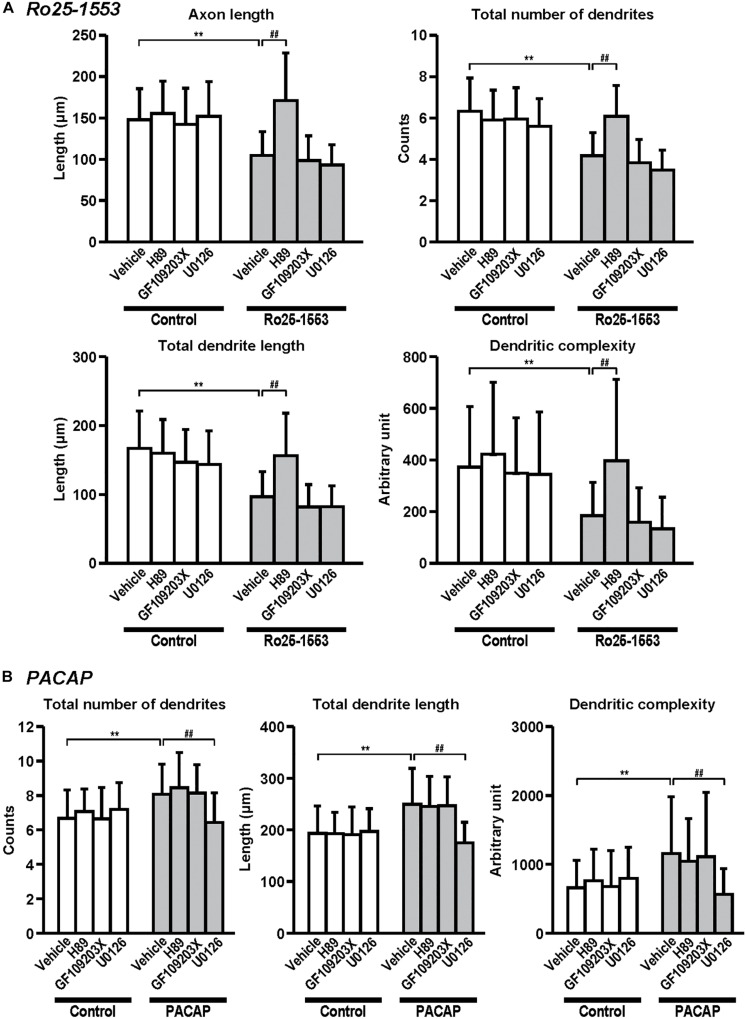
Differential signaling pathways in the effects of Ro25-1553 and PACAP on axon and dendritic outgrowth. Primary cortical neurons were cultured with Ro25-1553 (10 nM) for 3 days *in vitro* (DIV) **(A)** or PACAP (100 nM) for 7 DIV **(B)** and double-immunostained for pNF and MAP2. H89 (1 μM), GF109203X (1 μM), or U0126 (1 μM) was treated 30 min before the treatment with Ro25-1553 or PACAP. H89, but not GF109203X or U0126, blocked Ro25-1553-induced reductions in axon length (main effects: *F*_1,312_ = 55.022, *P* < 0.0001 for Ro25-1553, *F*_3,312_ = 21.421, *P* < 0.0001 for inhibitors; interaction: *F*_3,312_ = 14.153, *P* < 0.0001), total numbers (main effects: *F*_1,312_ = 107.531, *P* < 0.0001 for Ro25-1553, *F*_3,312_ = 17.086, *P* < 0.0001 for inhibitors; interaction: *F*_3,312_ = 14.788, *P* < 0.0001) and length (main effects: *F*_1,312_ = 93.817, *P* < 0.0001 for Ro25-1553, *F*_3,312_ = 16.605, *P* < 0.0001 for inhibitors; interaction: *F*_3,312_ = 9.139, *P* < 0.0001) of dendrites, and dendritic complexity (main effects: *F*_1,312_ = 39.122, *P* < 0.0001 for Ro25-1553, *F*_3,312_ = 10.089, *P* < 0.0001 for inhibitors; interaction: *F*_3,312_ = 3.091, *P* < 0.05). On the other hand, U0126, but not H89 or GF109203X, blocked PACAP-induced increases in total numbers (main effects: *F*_1,352_ = 23.759, *P* < 0.001 for PACAP, *F*_3,352_ = 4.738, *P* < 0.01 for inhibitors; interaction: *F*_3,352_ = 9.465, *P* < 0.0001) and length (main effects: *F*_1,352_ = 41.061, *P* < 0.001 for PACAP, *F*_3,352_ = 9.241, *P* < 0.0001 for inhibitors; interaction: *F*_3,352_ = 12.141, *P* < 0.0001) of dendrites, and dendritic complexity (main effects: *F*_1,352_ = 14.664, *P* < 0.001 for PACAP, *F*_3,352_ = 2.967, *P* < 0.05 for inhibitors; interaction: *F*_3,352_ = 6.756, *P* < 0.001). Values represent mean ± SD of 40–45 neurons from three independent experiments. **P < 0.01 vs. control, ^##^*P* < 0.01 vs. vehicle.

The pregnant ICR (CD1) mice at 16 days of gestation were purchased from Japan SLC, Inc (Hamamatsu, Japan). The generation of VPAC2 receptor deficient mice using gene targeting has been previously reported (Harmar et al., 2002). VPAC2 receptor homozygous knockout mice and littermate wild-type mice (C57BL/6 strain) used here were obtained by crossing with VPAC2 receptor heterozygous mice (Ago et al., 2017). Mice were housed in clear cages in groups of 3–5 animals under controlled environmental conditions (22 ± 1°C; 50 ± 10% relative humidity; a 12-h light-dark cycle, lights on at 0800 h; food and water *ad libitum*). Ro25-1553, VIP, and PACAP38 were purchased from Peptide Institute, Inc (Osaka, Japan). H89, GF109203X, and U0126 were purchased from Sigma-Aldrich (St. Louis, MO, United States). PG99-465 was purchased from Bachem (Bubendorf, Switzerland). The concentrations of the peptides and compounds used here were selected based on previous studies (Gourlet et al., 1997; Drahushuk et al., 2002; Leemhuis et al., 2007; Ogata et al., 2015). Primary neuronal cultures prepared from CD1 mice were used in most experiments (Figures 1–4, 6). Wild-type and VPAC2 receptor deficient C57BL/6 mice were used to examine the VPAC2 receptor-mediated effects of Ro25-1553 (Figure 5).

### Preparation of Primary Neuronal Cultures

Primary neuronal cultures were prepared from cerebral cortices of 16-day-old embryonic mice as previously described (Takuma et al., 2009; Kawanai et al., 2016). In brief, cerebral cortices were dissected from embryonic mice, sliced at a thickness of 0.5–1 mm with a razor blade under ice cold conditions (1–4°C), and incubated with 0.05% Trypsin-EDTA-4Na under a humidified atmosphere of 95% air/5% CO_2_ at 37°C for 15 min. Tissues were then mechanically dissociated with a fire-polished Pasteur pipette in Neurobasal^TM^ medium containing 2% B27 supplement, 2 mM L-glutamine, 50 units/mL penicillin, and 50 μg/mL streptomycin. Cells were then plated at a density of 1 × 10^4^ cells/cm^2^ on glass coverslips in 24-well tissue culture plates, which were coated with 20 μg/mL of poly-L-lysine (day 0). Cell cultures were kept at 37°C in a 95% air/5% CO_2_ humidified incubator. Cells were treated with Ro25-1553, VIP, or PACAP38 at 1 day *in vitro* (DIV) until 3, 7, or 14 DIV. During the experiments, the culture medium including freshly prepared compounds and peptides at the same concentration as 1 DIV was replaced every 3 days.

### Morphological Analysis

Cells were fixed with 4% paraformaldehyde for 10 min at 4°C, and incubated with 0.2% Triton X-100 in Ca^2+^-, Mg^2+^-free phosphate buffered saline (PBS, pH 7.2) for 5 min at room temperature. After blocking with 1% bovine serum albumin (BSA)/PBS for 30 min, they were incubated with a chicken polyclonal anti-MAP2 antibody (1:5000; Cat# ab5392, Abcam, Cambridge, United Kingdom; RRID:AB_2138153) and a mouse monoclonal anti-Neurofilament H & M, Phosphorylated antibody (1:500; Cat# SMI-310, Covance Japan, Tokyo, Japan; RRID:AB_448147) at 4°C overnight. After washing with PBS, the cells were incubated with species-specific fluorophore-conjugated secondary antibodies (1:200; Alexa 488-conjugated anti-chicken IgY (Cat# A32931, RRID:AB_2762843) and Alexa 594-conjugated anti-mouse IgG (Cat# A-11005, RRID:AB_2534073), Thermo Fisher Scientific) for 2 h at room temperature. The cells were washed with PBS and mounted using the ProLong^TM^ Gold antifade reagent (Thermo Fisher Scientific). Digitized images were obtained with an upright light microscope with a cooled CCD digital camera system (Axio Imager. M2/AxioCam MRc5; Carl Zeiss, Jena, Germany). In this study, phosphorylated neurofilaments (pNF)-positive neurites were classified morphologically as axons, and MAP2-positive and pNF-negative neurites were classified as dendrites. We exclude the cells that did not have axons or dendrites from further analysis. Additionally, a few complex overlapping neurons were not measured. Then, 10–20 neurons in each experiment were randomly selected by an observer blind to the treatment. Each experiment was independently repeated three times. The axon and all dendrites of each neuron were manually traced with Neurolucida software (Version 11; MBF Bioscience, Inc., Williston, VT, United States). The axon length, total number of dendrites (total number of terminal dendrite branches), total length of all dendrites, and dendritic complexity in each neuron were calculated from the traced neurites using Neurolucida. Dendritic complexity was assessed by measuring the lengths of dendrites and counting the number of branch points in each branch order. The dendritic complexity index was calculated from: (sum of the terminal orders + number of terminals) × (total dendritic length/number of primary dendrites) (Pillai et al., 2012). Terminal order indicates the number of sister branches emanating from the dendritic segment between a particular terminal tip and cell body.

### RT-PCR

Total RNAs from cultured cells were isolated using the SV Total RNA isolation system (Promega, Madison, WI, United States) according to the manufacturer’s instructions. The total RNAs were reverse transcribed with Superscript III (Thermo Fisher Scientific, Waltham, MA, United States). RT-PCR was performed with GoTaq^®^ Green Master Mix (Promega) using Applied Biosystems^TM^ Veriti^TM^ 96-Well Thermal Cycler. The following primers were used: 5′-ATGAGTCTTCCCCAGGTTG-3′ (forward) and 5′-ACCGACAGGTAGTAATAATCC-3′ (reverse) for the PAC1 receptor; 5′-AGTGAAGACCGGCTACACCA-3′ (forward) and 5′-TCGACCAGCAGCCAGAAGAA-3′ (reverse) for the VPAC1 receptor; 5′-ATGGACAGCAACTCGCCTC TCTTTAG-3′ (forward) and 5′-GGAAGGAACCAACACATAA CTCAAACAG-3′ (reverse) for the VPAC2 receptor; 5′-AC CACAGTCCATGCCATCAC-3′ (forward) and 5′-TCCA CCACCCTGTTGCTGTA-3′ (reverse) for GAPDH. PCR was performed for 40 cycles at 95°C for 30 s; 55°C for 30 s; and 72°C for 30 s.

### Statistical Analysis

All results are presented as the mean ± standard deviation (SD). Statistical analyses were performed using Statview (SAS Institute Japan Ltd., Tokyo, Japan), and significant differences determined by one- or two-way ANOVA followed by the Tukey–Kramer test. The threshold for statistical significance was defined as *P* < 0.05.

## Results

Figure 1A shows the gel images for RT-PCR for PAC1, VPAC1, VPAC2 receptors, and GAPDH, indicating that all VIP/PACAP receptors are expressed in primary cultured cortical neurons. Then, we examined the effects of VIP, Ro25-1553, and PACAP on axon and dendritic outgrowth in cultured cortical neurons. Figure 1B shows the representative pNF- and MAP2-immunostained images of primary cultured cortical neurons treated with Ro25-1553 (10 nM), a selective VPAC2 receptor agonist, VIP (10 nM), and PACAP (10 nM) for 3 days *in vitro* (DIV). Treatment with VIP (1, 10, and 100 nM) and Ro25-1553 (1–1000 nM) for 3 DIV significantly reduced axon length (Figure 1C). PACAP at doses used here (1–1000 nM) did not affect the axon length.

Figure 2 shows the effects of VIP on dendritic outgrowth. Primary cortical neurons were cultured with VIP for 14 DIV. Representative MAP2-immunostained images of neurons treated with different concentrations of VIP at 7 and 14 DIV are shown. With longer periods in culture, overall increase in dendrite extension and branching were observed. Quantitative morphological analysis revealed that VIP at doses of 1–100 nM, but not 1000 nM, decreased the total numbers and length of dendrites, and dendritic complexity for 3 to 14 DIV.

Figure 3 shows the effects of Ro25-1553 on dendritic outgrowth. Primary cortical neurons were cultured with Ro25-1553 for 14 DIV. Representative MAP2-immunostained images of neurons treated with different concentrations of Ro25-1553 at 7 and 14 DIV are shown. Quantitative morphological analysis revealed that Ro25-1553 at doses of 1–1000 nM decreased the total numbers and length of dendrites, and dendritic complexity for 3 to 14 DIV.

Figure 4 shows the effects of PACAP on dendritic outgrowth. Primary cortical neurons were cultured with PACAP for 7 DIV. Representative MAP2-immunostained images of neurons treated with different concentrations of PACAP at 7 DIV are shown. Quantitative morphological analysis revealed that PACAP (1–1000 nM) dose-dependently increased the total numbers and length of dendrites, and dendritic complexity for 3 to 7 DIV.

Figure 5 shows the involvement of the VPAC2 receptor in the inhibitory effects of Ro25-1553 and VIP on axon and dendritic outgrowth. Pretreatment with PG99-465 (100 nM), a VPAC2 receptor antagonist, blocked Ro25-1553 (10 nM)- and VIP (10 nM)-induced reductions in axon length, total numbers and length of dendrites, and dendritic complexity (Figure 5A). Additionally, Ro25-1553-induced reductions in axon length, total numbers and length of dendrites, and dendritic complexity were abolished in cortical neurons derived from VPAC2 receptor knockout mice (Figure 5B).

Figure 6 shows the signaling pathway involved in the effects of Ro25-1553 and PACAP on neurite outgrowth. Pretreatment with a PKA inhibitor H89 (1 μM), but not a PKC inhibitor GF109203X (1 μM) or a MEK inhibitor U0126 (1 μM), blocked Ro25-1553 (10 nM)-induced reductions in axon length, total numbers and length of dendrites, and dendritic complexity (Figure 6A). On the other hand, pretreatment with U0126, but not H89 or GF109203X, blocked PACAP (100 nM)-induced increases in total numbers and length of dendrites, and dendritic complexity (Figure 6B).

## Discussion

The present study confirmed that PAC1, VPAC1, and VPAC2 receptors are expressed in primary cultured mouse cortical neurons as observed *in vivo* mouse cortex (Marzagalli et al., 2016; Hirabayashi et al., 2018), indicating that Ro25-1553 and endogenous ligands VIP and PACAP could act through all VIP/PACAP receptors. We demonstrated that low concentrations (1–100 nM) of VIP and the VPAC2 receptor agonist Ro25-1553 reduced axon and dendritic outgrowth of cortical neural precursors measured at 3, 7, and 14 DIV. It is unlikely that the effects of Ro25-1553 and VIP on the numbers and complexity of axons and dendrites were due to general toxicity because the inhibitory effects of Ro25-1553 were abolished in VPAC2 receptor knockout mice (Figure 5B), and neither affected cell numbers based on the MTT [3-(4,5-dimethylthiazol-2-yl)-2,5-diphenyltetrazolium bromide] assay (data not shown). Interestingly, the effect of VIP was lost at higher concentration (1 μM), demonstrating a classical bell-shaped curve. This may be due to the fact that high dose of VIP can activate the PAC1 receptor (Drahushuk et al., 2002; Ogata et al., 2015), thereby producing an opposing effect which cancels the inhibitory action. On the other hand, PACAP, which binds with high affinity to PAC1 and VPAC2 receptors, promoted dendritic outgrowth of cortical neurons. In this context, the balance between VPAC2 receptor- and PAC1 receptor-mediated signals might be important to regulate the neurite outgrowth. However, we do not yet determine precisely why and how PACAP exhibits the opposite effect. To clarify this point, future studies on the effects of different concentrations of VIP and PACAP alone and in combination in either VPAC2- or PAC1-receptor knockout mice will be needed. Previously, Leemhuis et al. (2007) reported that VIP (1 nM) at 3 DIV for 18 h caused the increases in axon and dendritic outgrowth in cultured rat hippocampal neurons. This contrasts with the results of the present study using cortical neurons, again demonstrating that effects of these peptides are cell-type specific. Importantly, the effects of Ro25-1553 and VIP were blocked in the present study by pretreatment with a VPAC2 receptor antagonist PG99-465. Furthermore, we demonstrated the high specificity of the VPAC2 receptor agonist Ro25-1553, which was abolished in cortical neurons derived from VPAC2 receptor knockout mice. Overall, the results suggest that the activation of the VPAC2 receptor delays or limits the maturation in mouse cortical neurons. In a previous study, we also found that adult VPAC2 receptor knockout mice show abnormal dendritic morphology in the prelimbic and infralimbic cortices, but not basolateral amygdala (Ago et al., 2017). Loss of the VPAC2 receptor reorganized apical and basal dendritic arbors of prelimbic cortex neurons and apical, but not basal, dendritic arbors of infralimbic cortex neurons. In the prelimbic cortex neurons, the amount of apical dendritic material distal to the soma was decreased in VPAC2 receptor knockout mice, while proximal dendritic material was increased. In the infralimbic cortex, the amount of apical dendritic material proximal to the soma was increased in VPAC2 receptor knockout mice, while other indices of morphology did not differ. Although the present experimental design does not distinguish these regions, the findings suggest that the VPAC2 receptor plays an important role in regulating the development of dendritic morphology in the cerebral cortex. Like for PAC1 receptors, several splice variants of VPAC2 receptors in the mouse and human have been reported (Grinninger et al., 2004; Bokaei et al., 2006; Huang et al., 2006; Miller et al., 2006; Dickson and Finlayson, 2009) and there is a difference in the VPAC2 receptor expression level among brain regions (Sheward et al., 1995; Vertongen et al., 1997; Joo et al., 2004; Kalló et al., 2004; Marzagalli et al., 2016; Tian et al., 2019). These might explain at least partly the difference in the response to VIP in different neural cell types, but exact mechanisms remain unknown. It would be intriguing to examine whether activation of the VPAC2 receptor affects axon and dendritic outgrowth in different cell types.

Regarding the mechanism underlying the inhibitory effects of Ro25-1553 on cortical neuronal maturation, we found that a PKA inhibitor H89, but not a PKC inhibitor GF109203X or a MEK inhibitor U0126, prevented Ro25-1553-induced impairment of axon and dendritic outgrowth. This suggests that activation of the cAMP/PKA signaling pathway is involved in the VPAC2 receptor-induced impairment of axon and dendritic outgrowth. On the other hand, the opposing stimulatory effects of PACAP on dendritic outgrowth were blocked by U0126, but not H89 or GF109203X. Interestingly, PACAP38-induced ERK phosphorylation and neuritogenesis was MEK-dependent and PKA-independent in PC12-derived Neuroscreen-1 cells (Emery and Eiden, 2012). On the other hand, cAMP responsive element binding (CREB) phosphorylation induced by PACAP was blocked by H89 in these cells. These suggest that PACAP can stimulate two distinct and independent cAMP pathways. In contrast, Kambe and Miyata (2012) showed that PACAP at doses of 1 and 10 nM, but not 0.1 nM, increased neurite outgrowth in primary hippocampal neurons, and this effect was blocked by H89. Currently, the reason for the discrepancy about the involvement of PKA-mediated signaling in the effects of PACAP on neurite outgrowth is unknown. Together, these findings suggest that PACAP and VIP might have the diverse activities on neurite outgrowth via the distinct signaling pathway in different brain regions.

The impetus for our studies were the series of reports showing that micro-multiplications of the *VIPR2* gene is associated with schizophrenia (Levinson et al., 2011; Vacic et al., 2011; Yuan et al., 2014; Li et al., 2016; Marshall et al., 2017) and ASD (Vacic et al., 2011; Firouzabadi et al., 2017), and that these mutations were associated in patients with heightened VIP-induced cAMP responsiveness (Vacic et al., 2011). h*VIPR2*-BAC tg mice were recently shown to exhibit multiple psychiatric disorder-related behavioral phenotypes and early postnatal striatal developmental deficits that manifested as the elevated cAMP/PKA signaling, increased striatal excitatory inputs, and striatal dendritic maturation deficit (Tian et al., 2019). We also found that pharmacological activation of the VPAC2 receptor in the mouse during early postnatal development caused prepulse inhibition deficits and reductions in synaptic proteins synaptophysin and PSD-95 in the prefrontal cortex (Ago et al., 2015). Impairments of dendritic and synaptic density in pyramidal neurons across multiple brain regions, such as changes in dendritic arborization, dendritic spine number/type, and morphology, have been observed in schizophrenia (Moyer et al., 2015). In particular, reduced length of basilar dendrites and reduced dendritic number have been found in layer 3 in the prefrontal cortical areas (Brodmann area (BA) 10, BA 11, and BA 46), anterior cingulate cortex (BA 32) of schizophrenic patients (Glantz and Lewis, 2000; Kalus et al., 2000; Broadbelt et al., 2002; Black et al., 2004; Konopaske et al., 2014). VIP has been known to be highly expressed in the layer 2/3 inhibitory interneurons, and thus VIP neurons control neocortical activity (Tremblay et al., 2016). Taken together, the present *in vitro* study suggests that the activation of the VPAC2 receptor directly disrupts cortical neuronal maturation and implies that the *VIPR2* linkage can be explained in part by impaired neuronal maturation due to overactivity of VPAC2 receptors at a time of brain development when neural circuits involved in cognition and social behavior are being established and/or that VPAC2 receptor overactivity disrupts ongoing synaptic plasticity in the adult brain.

## Data Availability Statement

The datasets generated for this study are available on reasonable request to the corresponding author.

## Ethics Statement

All animal studies were approved by the Animal Research Committee at University of California, Los Angeles (UCLA) and the Animal Care and Use Committee of the Graduate School of Pharmaceutical Sciences, Osaka University, and the Graduate School of Biomedical and Health Sciences, Hiroshima University. All experimental procedures were conducted in accordance with the guidelines of the *Guide for the Care and Use of Laboratory Animals* (National Research Council, 1996). Every effort was made to minimize animal suffering, and to reduce the number of animals used.

## Author Contributions

ST, TK, RY, LC, TM, MY, SA, AH-T, and YA performed the experiments and analyzed the data. TN, KY, NH, SN, KT, JW, HH, and YA supported the study, designed study, and wrote the manuscript. HH and YA reviewed and approved the manuscript and held all the responsibilities related to this manuscript. All authors reviewed and approved the manuscript.

## Conflict of Interest

The authors declare that this study received funding from Shionogi & Co., Ltd. The funder had the following involvement with the study: design of the study. KY and NH are full-time employees of Shionogi & Co., Ltd.
